# Clinical and Dermoscopic Evaluation of Patients With Topical Steroid Damaged Faces (TSDF)

**DOI:** 10.7759/cureus.74624

**Published:** 2024-11-27

**Authors:** Arushi Goel, Aneet Mahendra, Sanjeev Gupta

**Affiliations:** 1 Dermatology, Maharishi Markandeshwar Institute of Medical Sciences and Research, Ambala, IND

**Keywords:** dermoscopy, facial dermatoses, side effects, telangiectasia, topical corticosteroids (tcs), topical steroid damaged face (tsdf)

## Abstract

Background

Topical steroid-dependent or damaged face (TSDF) is a common condition where the widespread availability of over-the-counter topical corticosteroid (TCS)-containing products leads to their misuse and addiction. Prolonged use of these steroids on the face can result in significant side effects. Early diagnosis and cessation of steroid use are important. Dermoscopy can serve as an easy-to-use and non-invasive tool for the early diagnosis and for assessing the exact extent of damage caused by steroid misuse.

Aims and objectives

To observe the various clinical and demographic features of patients with TSDF, the dermoscopic features of these patients, and to find their association with the potency and duration of application of TCS.

Material and methods

In this observational, cross-sectional study of 250 patients (aged 12-60 years, both genders) presenting with facial dermatosis and clinical signs of TSDF, having a history of at least one month of TCS use, were enrolled using consecutive sampling. Demographic data, clinical features, and dermoscopic findings were recorded. Dermoscopic results were compared with clinical examination, TCS potency, and duration of application.

Results

The majority (208; 83.2%) of the patients were females, and most of them belonged to the age group of 26-35 years (87; 34.8%). Hyperpigmentation (101; 40.4%) was the most prevalent indication for TCS use in the study. The most common clinical findings included erythema, hypertrichosis, and telangiectasia. Dermoscopic findings such as linear, serpentine, polygonal, and Y-shaped vessels, along with breaking of the pseudo reticular network, red diffuse areas, Demodex tails, and comedones were significantly associated with the duration of TCS use. Linear vessels, Demodex tails, and comedones also correlated significantly with TCS potency, while most other findings did not.

Conclusion

Dermoscopy is a novel, non-invasive diagnostic technique for the early identification of TCS abuse. In TSDF, it confirms the diagnosis and predicts disease severity earlier than examination by the naked eye alone.

## Introduction

Topical corticosteroids (TCS) have been widely used in dermatology for over 50 years, starting with their introduction by Sulzberger MB and Witten VH in 1952 [1]. Since then, steroid molecules with varying potencies have emerged, enabling the treatment of various inflammatory dermatoses [2]. Besides their anti-inflammatory effects, they possess antipruritic, immunosuppressive, and melanopenic properties.

Although immensely beneficial, TCS have been widely abused by quacks, unqualified individuals, and the general public [2]. In India, TCS are readily available as over-the-counter (OTC) drugs sold under misleading names. This has led to widespread misuse, resulting in a range of adverse effects known as "topical steroid-damaged face," as noted by Lahiri K and Coondoo A in 2008 [2].

The acronym "TSDF" describes the semi-permanent or permanent damage inflicted on facial skin due to the irrational, unchecked, indiscriminate, or prolonged application of TCS, leading to multiple cutaneous symptoms and signs [2]. This condition typically occurs from the prolonged use of potent topical steroids on the face, exceeding recommended durations or concentrations, or using them for non-indicated conditions like acne or cosmetic purposes. The face suffers the most from this potentially hazardous therapy as the skin on the face is relatively thinner and more sensitive. The facial epidermis (0.12 mm) is comparatively thinner than that of the rest of the body (0.60 mm), which results in increased percutaneous absorption of drugs.

Among the common adverse effects of TCS abuse are skin thinning (atrophy), erythema, telangiectasia, hypertrichosis, dyspigmentation, and acneiform eruptions [3]. The clinical presentation of TSDF results from a combination of dermal atrophy (due to TCS inhibiting collagen and the synthesis of hyaluronic acid by fibroblasts), inhibition of the action of nitric oxide (NO), and local immunosuppression. Upon withdrawal of TCS, endothelial NO is released, causing vasodilation and subsequent erythema.

Prolonged misuse of TCS on the face requires time to heal. Hence, early diagnosis and discontinuation of steroid use are important for effective treatment. Dermoscopy may serve as an easy-to-use tool for diagnosis and assessing the extent of damage from TCS abuse.

A dermoscope is a hand-held device with an inbuilt light source that magnifies a lesion and reduces the interference of light reflected from the skin surface. This enables the visualization of vascular and pigmented structures located in the deeper layers of the epidermis and the superficial parts of the dermis. It allows the exploration of features and characteristics beneath the skin that may not be visible to the naked eye. Additionally, the images captured with a dermoscope can be digitally photographed and recorded for future reference or use.

## Materials and methods

Study design

An observational, cross-sectional study design was adopted.

Study area

The study included patients with facial dermatosis exhibiting clinical signs and symptoms of TSDF who had a history of TCS application for at least one month. These patients presented to the Dermatology Outpatient Clinic at Maharishi Markandeshwar Institute of Medical Sciences and Research (MMIMSR), Mullana, Ambala, India. The study was conducted over a 12-month period from 2023 to 2024.

Study population and sample size

The study included 250 patients, of either gender, aged between 12 and 60 years. Patients taking oral corticosteroids for any reason and those with comorbidities such as thyroid disorders, Cushing’s syndrome, rosacea, or polycystic ovaries were excluded.

Ethical considerations

Institutional Ethics Committee approval was obtained prior to the commencement of the study (IEC-2546). Patients were enrolled after receiving an in-depth explanation about the nature of the study, and informed consent was obtained, reassuring confidentiality of the information shared.

Study measures

Data collected included demographic details, chief presenting complaints, and the source of the drug, along with indications, frequency, potency, and duration of TCS application. Cutaneous unaided examination of the patients was followed by dermoscopic evaluation using a Dermoscope AF 4515ZT (Dinolite), and images were captured with an iPhone 15 Pro (Apple Inc.). Dermoscopic findings were compared with TCS potency [4] and duration of use. Lastly, cutaneous examination results were compared with dermoscopic findings.

Statistical analysis

Statistical analysis was conducted using the Chi-square test and Fischer’s exact test, with significance levels set at p < 0.05 and a 95% CI.

## Results

**Figure 1:**
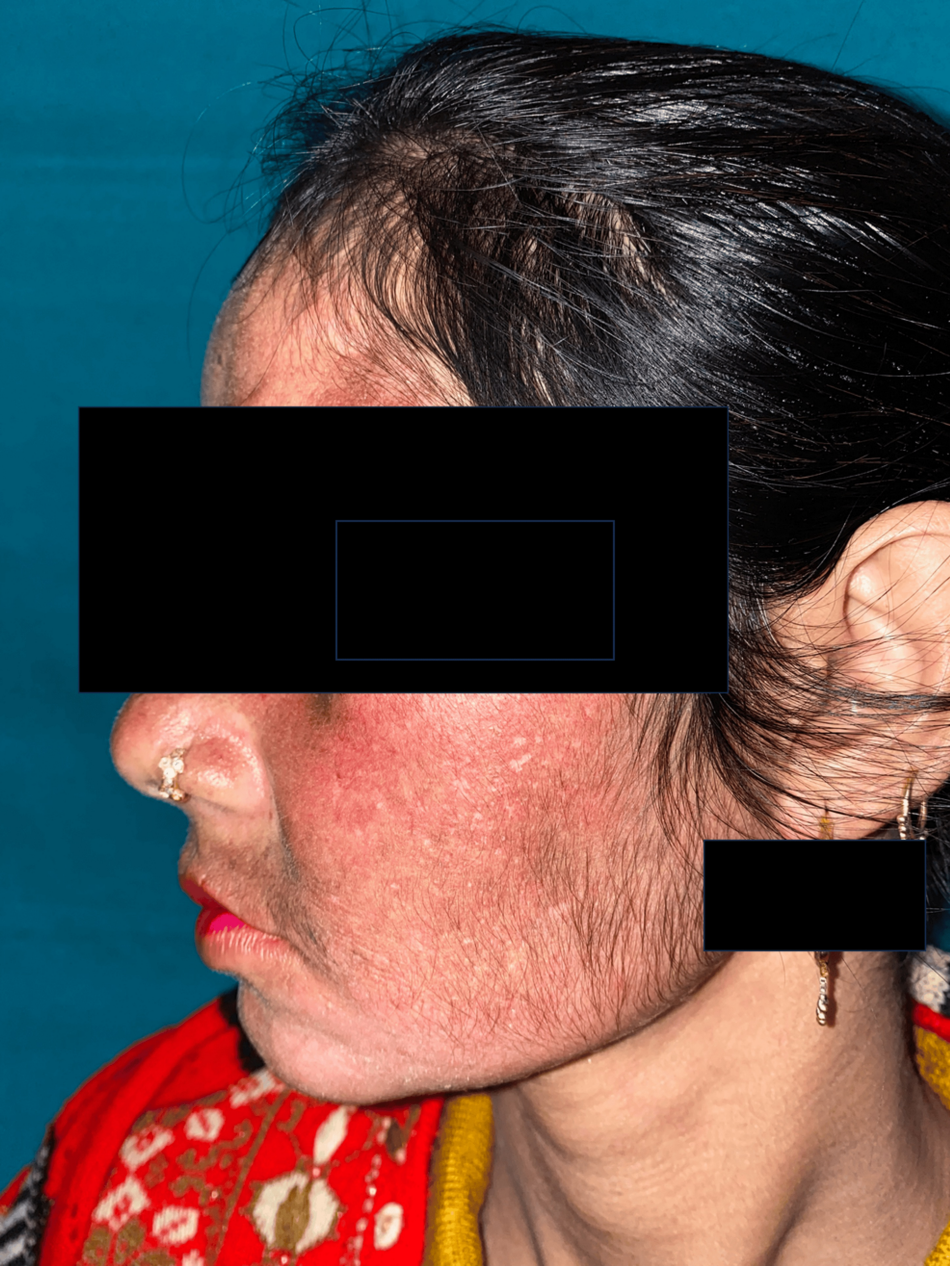
Clinical picture of a TSDF patient showing erythema, hyperpigmentation, and hypertrichosis. TSDF: Topical steroid-dependent or damaged face.

**Figure 2:**
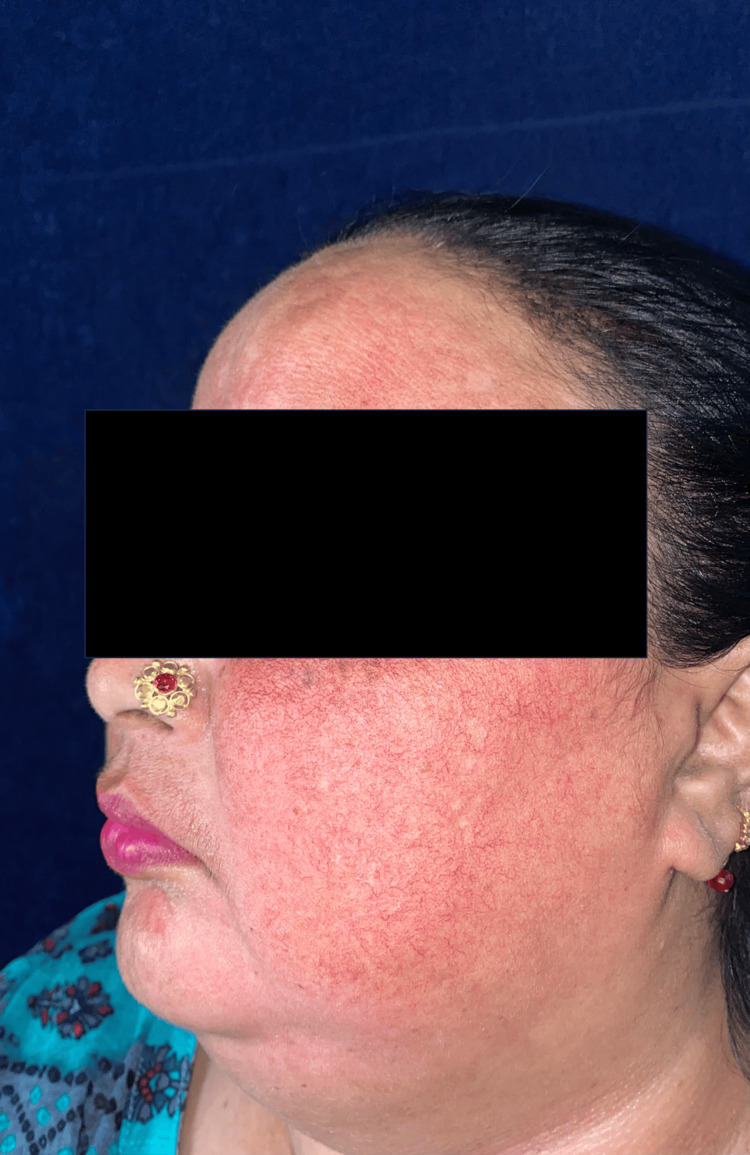
Clinical picture of a TSDF patient showing erythema and telangiectasia. TSDF: Topical steroid-dependent or damaged face.

**Figure 3:**
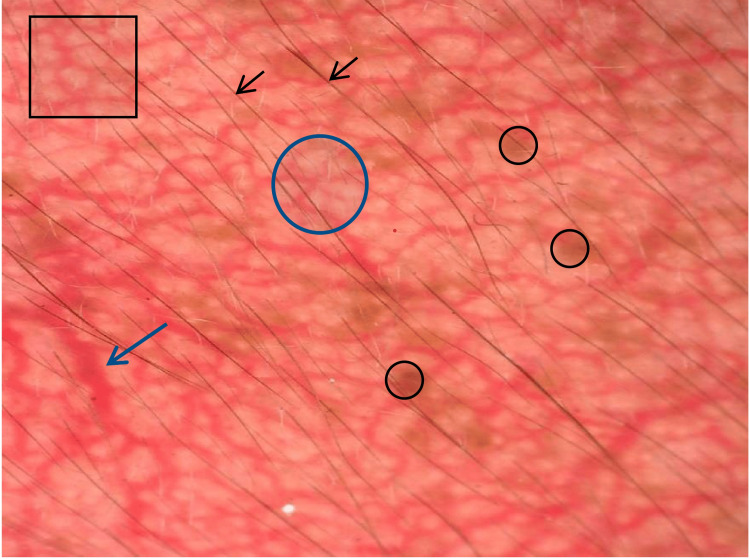
Dermoscopic picture showing red diffuse areas, brown globules (black circle), hypertrichosis (black arrow), polygonal vessels (black square), serpentine vessels (blue arrow), and branched vessels (blue circle).

**Figure 4:**
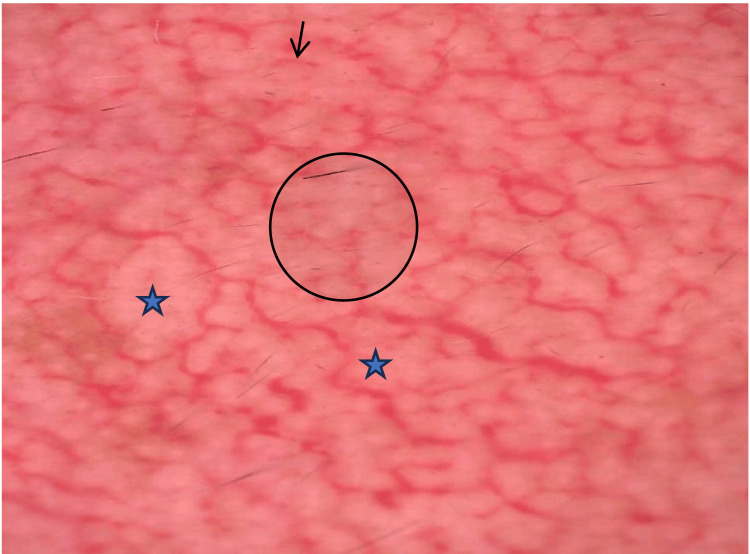
Dermoscopic picture showing breaking of the pseudoreticular network (black circle), white structureless areas (blue star), and linear vessels (black arrow).

**Figure 5:**
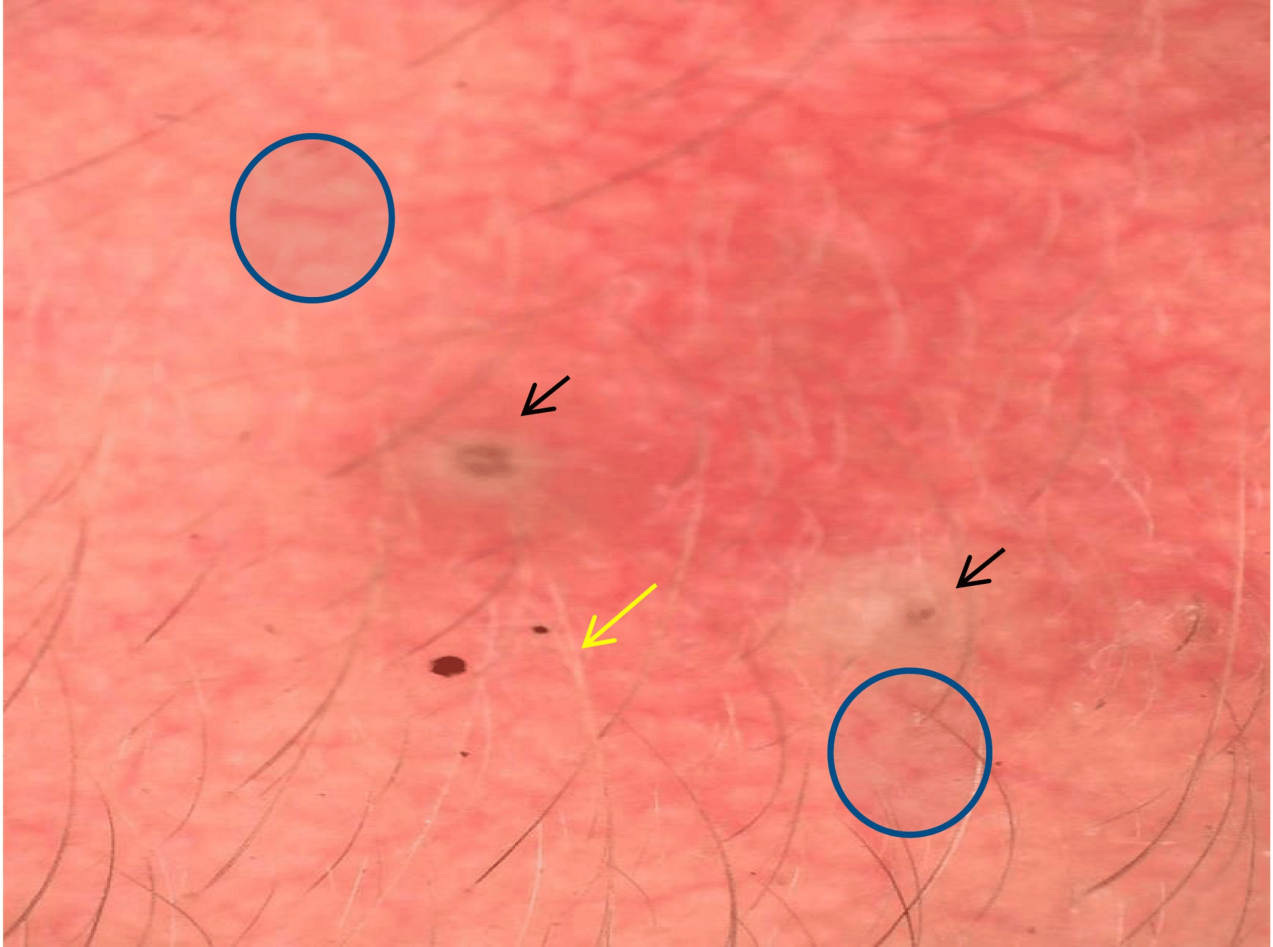
Dermoscopic picture showing fine vessels (blue circle), a comedone (black arrow), and a white hair (yellow arrow).

**Figure 6:**
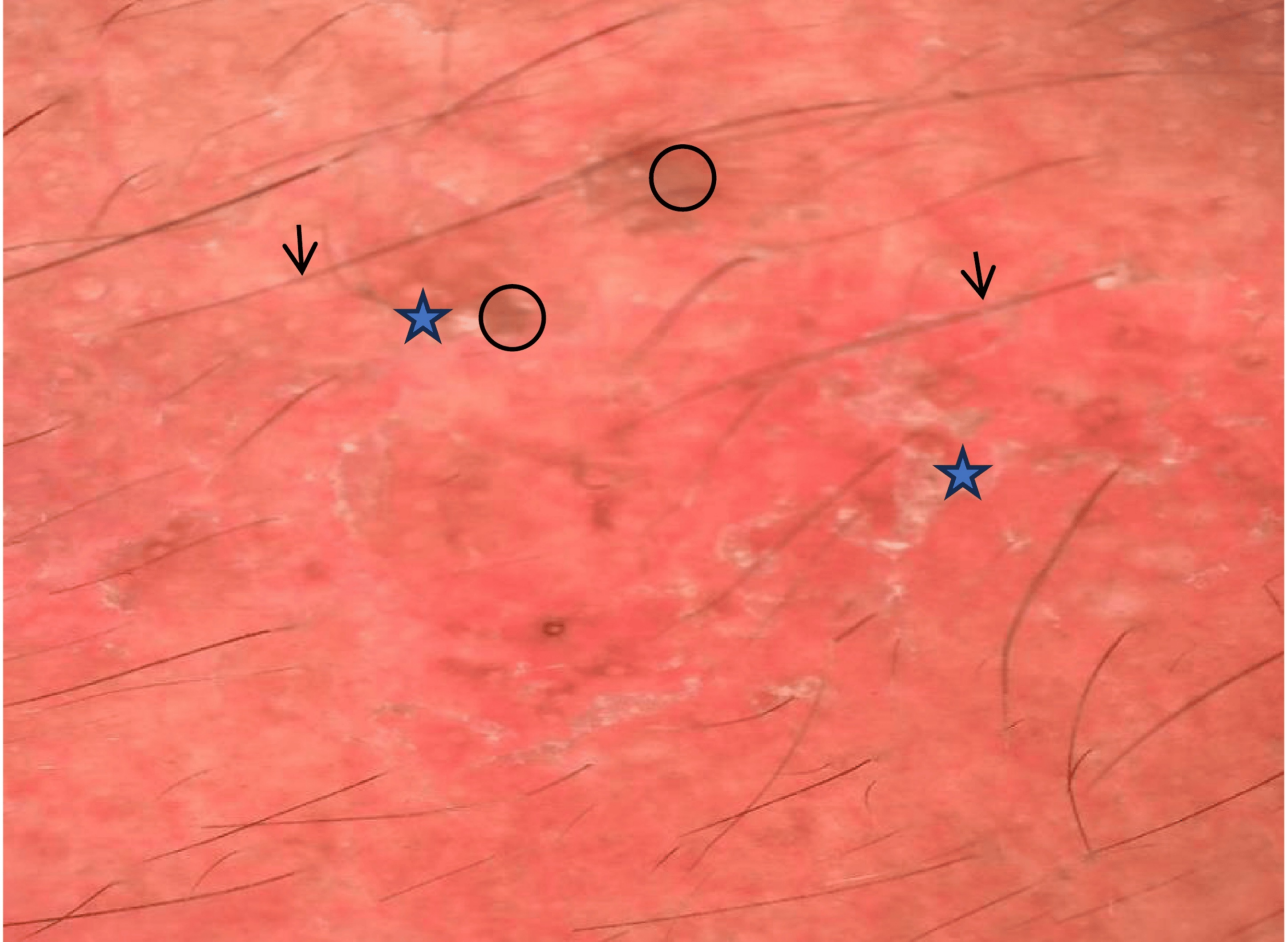
Dermoscopic picture showing desquamation (blue star), red diffuse areas, brown globules (black circle), and hypertrichosis (black arrow).

**Figure 7:**
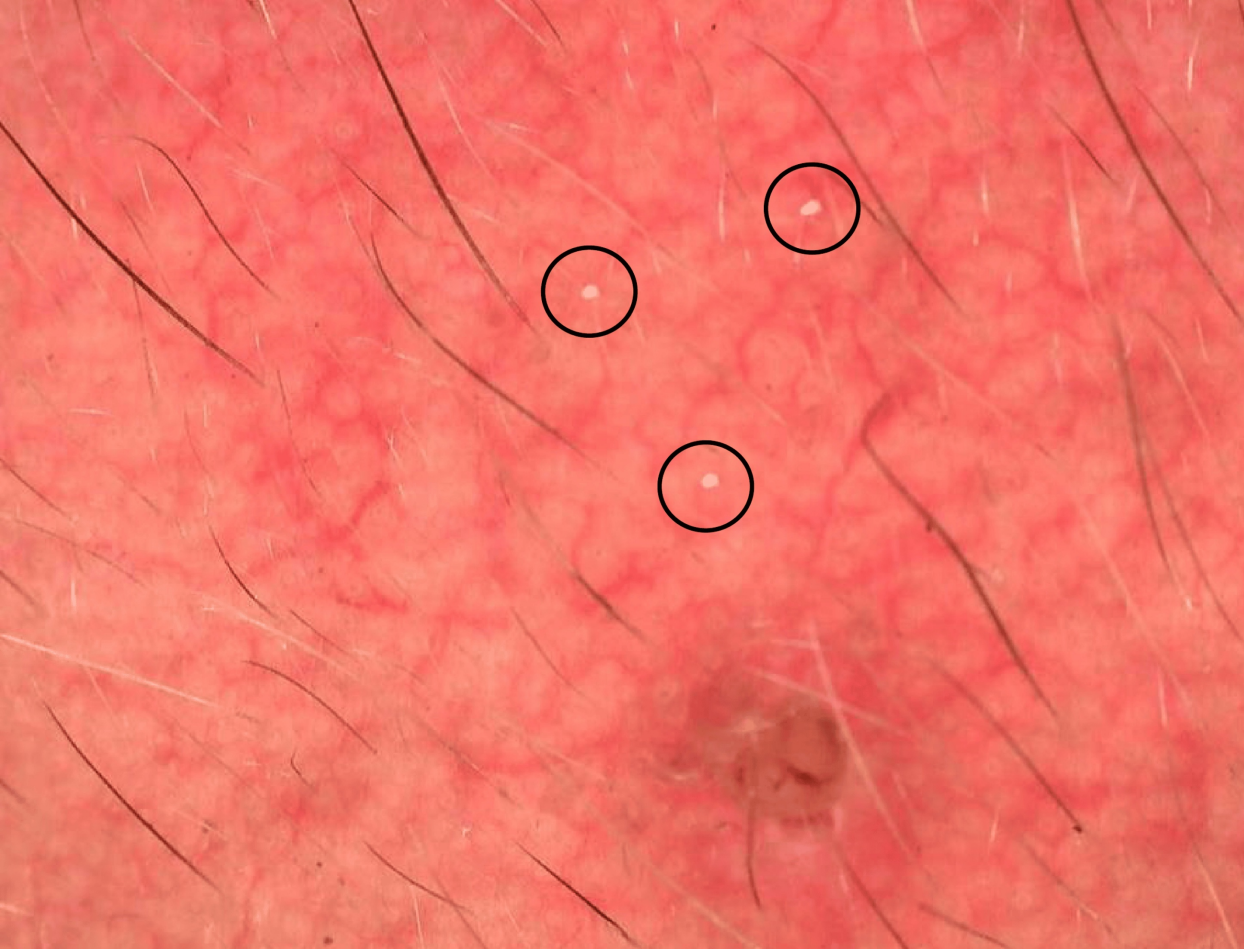
Dermoscopic picture showing Demodex tails (black circle).

**Figure 8:**
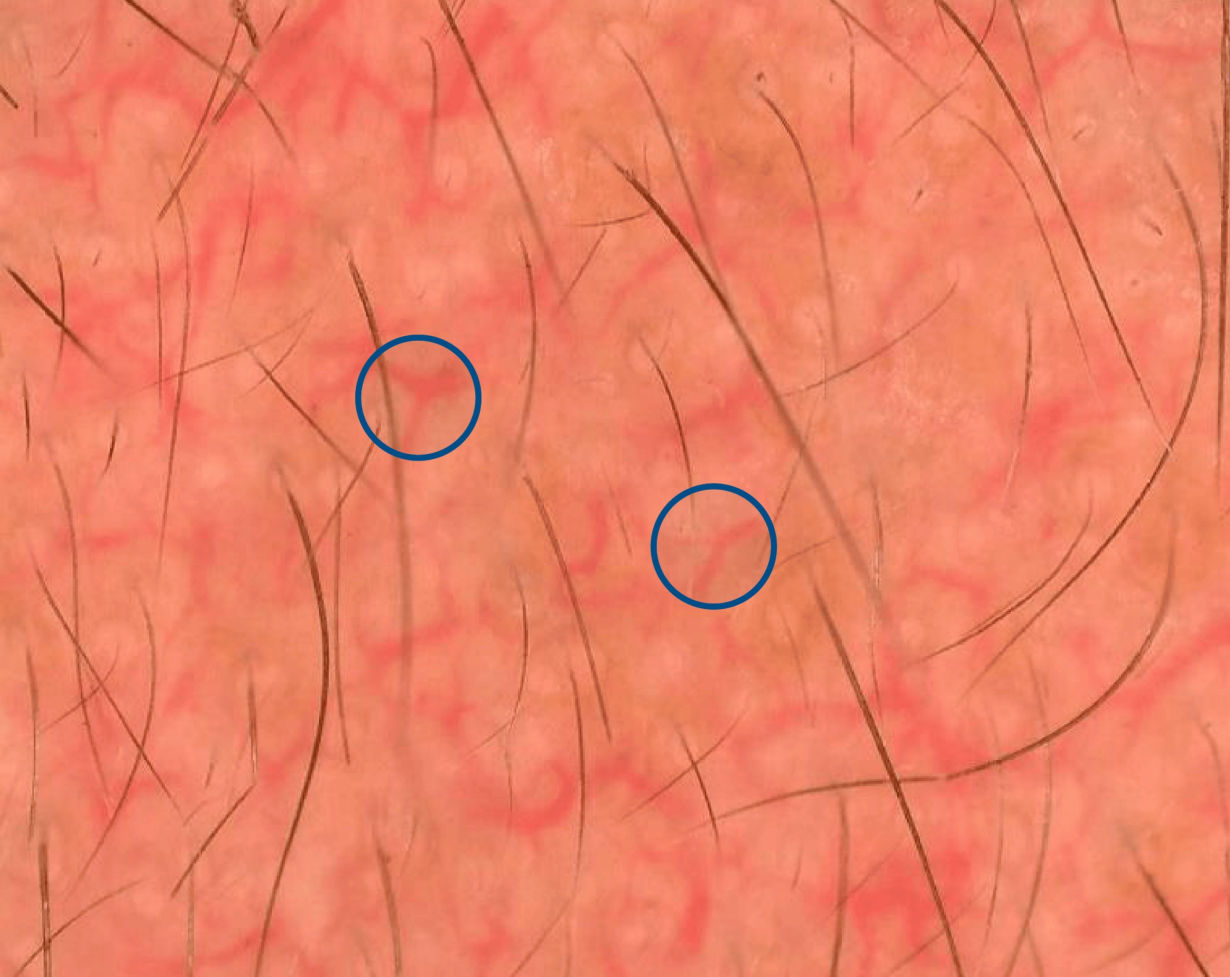
Dermoscopic picture showing Y-shaped vessels (blue circle).

A total of 250 patients were included in the study, of which the majority (87; 34.8%) belonged to the 26-35 age group with a mean age of 32.88 years. Most of the patients were females, constituting 208 (83.2%) with a male-to-female ratio of 1:4.95. Most patients belonged to rural areas, accounting for 187 (74.8%). One hundred eighty-three (73.2%) patients had received some form of formal education, while 67 (26.8%) were illiterate (Table 1). Chief complaints in the study varied from redness (182; 72.8%), photosensitivity (99; 39.6%), facial hair (96; 38.4%), pigmentation (94; 37.6%) to comedones (7; 2.8%) and white scars (7; 2.8%). The majority of the patients presented with more than one chief complaint. Some of the common indications for TCS application were hyperpigmentation disorders like melasma (101; 40.4%), as fairness cream (48; 19.2%), acne (37; 14.8%), and fungal infection (29; 11.6%). One hundred fifty-six (62.4%) patients used TCS once daily, 91 (36.4%) twice daily, and only 3 (1.2%) thrice daily. Class I potency TCS (85; 34%) were most commonly used, and class VI (5; 2%) were least used. The majority of the patients applied TCS for more than 12 months (118; 47.2%). In the study, the majority used TCS upon the recommendation of relatives, i.e., 92 patients (36.8%). On clinical examination, the most common finding was erythema (214; 65.6%) followed by hypertrichosis (156; 62.4%), telangiectasia (128; 51.2%), and hyperpigmentation (101; 40.4%) (Figures 1-2). The dermoscopic findings (Figures 3-8) seen in study subjects were categorized broadly into four groups: vascularity, background, adnexa, and others. In the vascularity group, the most common types of vessels seen upon dermoscopy were branched vessels (178; 71.2%) and serpentine vessels (139; 55.6%). Most patients showed red diffuse areas (236; 94.4%) and brown globules (195; 78%) in the background domain. In the adnexa group, hypertrichosis (211; 84.4%) was the most common finding and in others, papules (72; 28.8%) were commonly seen. There was a statistically significant association in the appearance of dermoscopic findings with the duration of TCS application, namely linear vessels, serpentine vessels, polygonal vessels, Y-shaped vessels, breaking of the pseudoreticular network, red diffuse areas, Demodex tails, and comedones (Table 2). Dermoscopic findings were also studied in association with the potency of TCS, in which linear vessels (p = <0.001), Demodex tails (p = 0.04), and comedones (p = 0.02) had a significant association. No other dermoscopic findings showed a significant association with the potency of TCS used (Table 3). Comparing cutaneous findings of erythema with dermoscopic findings of red diffuse areas showed statistical significance (p = 0.001). Similarly, other dermoscopic findings such as vessels, hypertrichosis, white hairs, brown globules, white structureless areas, desquamation, and papules were found in more patients compared to those showing corresponding findings upon inspection with the naked eye (Table 4).

**Table 1:** Demographic characteristics of study subjects (n=250). TCS: Topical corticosteroid.

Age group	No.	%
12-25 years	68	27.2
26-35 years	87	34.8
36-45 years	66	26.4
46-55 years	24	9.6
>55 years	5	2.0
Sex	No.	%
Female	208	83.2
Male	42	16.8
Educational status	No.	%
Elementary school	112	44.8
Graduate	9	3.6
High school	62	24.8
Illiterate	67	26.8
Duration of TCS application	No.	%
Upto 6 months	30	12.0
6-12 months	102	40.8
>12 months	118	47.2
Source of drug recommendation	No.	%
Beautician	5	2.0
Friend	21	8.4
Local pharmacist	73	29.2
Local practitioner	18	7.2
Neighbour	18	7.2
Relative	92	36.8
Self	23	9.2

**Table 2:** Association of the duration of topical steroid application with dermoscopic findings in study subjects (n=250). The Chi-square test was used for statistical significance. P < 0.05 is considered significant.

	Upto 6 months (n=30)	6-12 months (n=102)	>12 months (n=118)	P-value
Vascularity				
Linear	2 (6.7%)	38 (37.3%)	86 (72.9%)	<0.001
Serpentine	4 (13.3%)	37 (36.3%)	98 (83.1%)	<0.001
Polygonal	8 (26.7%)	20 (19.6%)	71 (60.2%)	<0.001
Fine	5 (16.7%)	17 (16.7%)	16 (13.6%)	0.79
Branched vessels	16 (53.3%)	76 (74.5%)	86 (72.9%)	0.06
Y-Shaped vessels	2 (6.7%)	20 (19.6%)	39 (33.1%)	<0.01
Background				
Breaking of pseudoreticular network	20 (66.7%)	64 (62.7%)	99 (83.9%)	0.001
White structureless areas	15 (50.0%)	61 (59.8%)	70 (59.3%)	0.6
Brown globules	26 (86.7%)	72 (70.6%)	97 (82.2%)	0.05
Red diffuse areas	27 (90.0%)	91 (89.2%)	118 (100.0%)	0.001
Desquamation	8 (26.7%)	21 (20.6%)	20 (16.9%)	0.46
Adnexa				
Follicular plugging	2 (6.7%)	18 (17.6%)	13 (11.0%)	0.18
Demodex tails	0 (0.0%)	5 (4.9%)	25 (21.2%)	<0.001
Comedones	6 (20.0%)	16 (15.7%)	7 (5.9%)	0.02
Hypertrichosis	25 (83.3%)	84 (82.4%)	102 (86.4%)	0.69
White hair	2 (6.7%)	19 (18.6%)	20 (16.9%)	0.29
Others				
Papules	9 (30.0%)	32 (31.4%)	31 (26.3%)	0.69
Pustules	6 (20.0%)	20 (19.6%)	15 (12.7%)	0.33

**Table 3:** Association of the potency of topical steroids with dermoscopic findings in study subjects (n=250). The Chi-square test or Fisher’s exact test was used for statistical significance. P < 0.05 is considered significant.

	Class I-III potency TCS (n=188)	Class IV and above potency TCS (n=62)	P-value
Vascularity			
Linear	81 (43.1%)	45 (72.6%)	<0.001
Serpentine	100 (53.2%)	39 (62.9%)	0.18
Polygonal	70 (37.2%)	29 (46.8%)	0.18
Fine	27 (14.4%)	11 (17.7%)	0.52
Branched vessels	138 (73.4%)	40 (64.5%)	0.18
Y-Shaped vessels	41 (21.8%)	20 (32.3%)	0.09
Background			
Breaking of pseudoreticular network	135 (71.8%)	48 (77.4%)	0.38
White structureless areas	109 (58.0%)	37 (59.7%)	0.81
Brown globules	144 (76.6%)	51 (82.3%)	0.35
Red diffuse areas	176 (93.6%)	60 (96.8%)	0.52
Desquamation	40 (21.3%)	9 (14.5%)	0.27
Adnexa			
Follicular plugging	25 (13.3%)	8 (12.9%)	1
Demodex tails	27 (14.4%)	3 (4.8%)	0.04
Comedones	27 (14.4%)	2 (3.2%)	0.02
Hypertrichosis	159 (84.6%)	52 (83.9%)	1
White hair	32 (17.0%)	9 (14.5%)	0.69
Others			
Papules	51 (27.1%)	21 (33.9%)	0.3
Pustules	32 (17.0%)	9 (14.5%)	0.69

**Table 4:** Comparison of cutaneous findings and corresponding dermoscopic findings in study subjects (n=250). The Chi-square test or Fisher’s exact test was used for statistical significance. P < 0.05 is considered significant.

Cutaneous findings			Corresponding Dermoscopic findings			P-value
	No.	%		No.	%	
Erythema	214	85.6	Red diffuse areas	236	94.4	0.001
Telengiectasia	128	51.2	Vessels (linear, serpentine, polygonal, fine, branched, Y-shaped)	226	90.4	<0.001
Papular eruption	51	20.4	Papules	72	28.8	0.03
Pustular eruption	34	13.6	Pustules	41	16.4	0.45
Comedones	20	8	Comedones	29	11.6	0.22
Hyperpigmenation	101	40.4	Brown globules	195	78	<0.001
Hypertrichosis	156	62.4	Hypertrichosis	211	84.4	<0.001
White Hair	20	8	White Hair	41	16.4	<0.01
Scaling	23	9.2	Desquamation	49	19.6	<0.001
Atrophy	14	5.6	White structureless areas	146	58.4	<0.001

## Discussion

In India, the unsupervised use of TCS is becoming a growing problem. Patients often start using TCS for minor skin issues like acne or melasma, based on recommendations from friends, family, or local pharmacists. Initially, the anti-inflammatory and vasoconstrictive properties of steroids seem to improve the appearance of the underlying skin issue. However, continuous use eventually results in epidermal thinning, dermal breakdown, and collagen damage [5]. In India, many potent TCS are classified as Schedule H drugs, meaning they can only be sold with a prescription from a registered medical practitioner. While the laws exist, enforcement remains a challenge, particularly in rural areas or small towns where pharmacies may sell potent TCS without a prescription. Cultural beauty standards emphasize flawless skin, pressuring young individuals to seek quick fixes. Additionally, there is a lack of public awareness regarding proper TCS use, leading to frequent misuse. The prolonged and repeated use of TCS has resulted in the manifestation of dermatoses on the face, referred to as TSDF [2].

Young patients tend to be more concerned about their appearance due to being in a stage of life where they are focused on marriage and career prospects, making them vulnerable to use TCS incessantly in pursuit of a fairer and more radiant complexion. Past studies indicate the most common age group to be 26-35 years, consistent with the findings in the present study [6-7].

The higher prevalence among females, noted in this study, is likely due to greater concerns about appearance, societal pressures, and the misconception that TCS are skin-lightening creams.

Social media influencers significantly contribute to the irrational rise in the use of TCS on the face. Many influencers lack formal dermatological training, which can result in the spread of misinformation. They might share personal anecdotes or unverified claims about TCS efficacy, convincing followers that these products are safe for long-term use.

It is a common perception that educated individuals are aware of TCS side effects, while misuse is more prevalent among the uneducated. However, misuse can occur across all educational levels due to inadequate medical advice, misinformation, or lack of healthcare access. In our study, only 67 (26.8%) were illiterate, while the rest had received some form of formal education, consistent with the findings by Dey [8]. Hence, the educated lot can also act ignorantly and can get entangled in the swamp of continuous TCS usage without supervision.

Pharmaceutical marketing that associates clear skin with beauty and success can pressure individuals to seek quick fixes, such as TCS. Many companies incorporate corticosteroids into their broader skincare product lines, marketing them as part of everyday beauty routines instead of positioning them as targeted treatments for specific conditions.

The presenting complaints in this study spanned from redness, photosensitivity, facial hair, itching, burning to pigmentation and acne. Mechanisms like rebound dilation of the blood vessels, accumulation of nitric oxide, and cytokine release were thought to be accountable for the occurrence of itching, redness, and burning sensation.

More than half of the patients (131; 52.4%) used TCS upon the recommendation of relatives, friends, and neighbors. Many patients (73; 29.2%) got it from local pharmacists, consistent with the study by Mamatha P et al. [9], thus taking advantage of the over-the-counter availability of TCS. The sequence of events leading to steroid abuse starts when a patient benefits from a potent TCS prescribed by a doctor for some other condition and, flattered by the response, the patient continues to use it and often recommends it to others as well. The ultimate victims of TCS misuse are the general public, including patients and those driven by beauty and fairness trends promoted by the beauty industry.

Many people perceive TCS as a universal remedy and apply them to any skin rash without seeking professional advice. Most of our patients used TCS of Class I, II, and III, which was in concordance with past studies [6-7,9]. This could be attributed to the fact that these steroids, like cream formulations of clobetasol propionate and betamethasone valerate, are the most cost-effective and easily available among all. A significant number of our patients disclosed the use of double or triple combination creams containing an antibiotic, an antifungal, and a TCS.

The most common findings upon clinical examination were erythema, hypertrichosis, and telangiectasia. Sethi S et al. [3] noted erythema as the most common clinical feature in TSDF patients, while Tatu [10] found telangiectasia to be the most frequent finding.

Dermoscopy has emerged as a valuable modality to evaluate minor skin changes and can be very useful in patients with TSDF. It can identify early signs of TCS abuse that may not be well appreciated with a naked eye examination. Thus, dermoscopy in TSDF can help in many ways, from confirmation of the diagnosis to differentiation from other causes of a red face. Additionally, dermoscopy significantly enhances communication between doctors and patients. By displaying the dermoscopic image of a lesion, it becomes easier to explain the diagnosis to patients [3]. This visual aid also helps patients grasp the severity of topical steroid abuse, as images paired with simple explanations can overcome the common denial of TCS usage despite frequent dermatologist inquiries. Often, this approach reduces ongoing steroid misuse and improves adherence to treatment.

In the study by Sethi S et al. [3], dermoscopic findings such as red diffuse areas, brown globules, various types of vessels, and hypertrichosis were in concordance with our study. Red diffuse areas were also frequently observed during dermoscopic examination in the study by Mamatha P et al. [9].

The evaluation of the association between the duration of TCS application and dermoscopic findings showed significant correlations. Notable findings included linear, serpentine, polygonal, and Y-shaped vessels, breaking of the pseudoreticular network, red diffuse areas, Demodex tails, and comedones (Table 2). As the duration of TCS application increases, most dermoscopic findings tend to progress. In the study by Sethi S et al. [3], statistical significance was achieved for Y-shaped vessels and brown globules with TCS use exceeding 3 months, and for polygonal vessels with TCS use exceeding 6 months.

The current study also examined the association between TCS potency and dermoscopic findings. It was observed that only linear vessels, Demodex tails, and comedones were statistically significant and more prevalent in patients using Class I, II, and III TCS (Table 3). Ankad BS et al. [11] found significant associations with TCS potency in features like red diffuse areas, white hair, and focal white areas. In contrast, Sethi S et al. [3] used a similar grouping method as ours but found no significant associations in their study.

In our study, a comparison of cutaneous and dermoscopy findings revealed that the majority of findings showed statistical significance, implying that corresponding findings can be seen in a higher number of patients when seen on dermoscopic examination as compared to naked eye examination alone (Table 4).

Thereby, dermoscopy can be used not only for early diagnosis but also for therapeutic decision-making and above all acts as a piece of objective evidence helping to convince patients about the detrimental effects of TCS.

Through this study, we wish to highlight that dermoscopy serves to non-invasively confirm suspicions and also aids in enhancing patient comprehension regarding the severity of topical steroid abuse by illustrating images explained in patient-friendly terms. This approach is anticipated to mitigate further steroid misuse and enhance treatment adherence.

Duration of TCS use may have been approximate rather than being exact, lack of histopathological correlation, and small sample size are the limitations of our study.

## Conclusions

TCS have been widely misused over the years in various ways. TSDF is the term given to the ill effects caused by abuse of TCS on the face. Dermoscopy is a novel non-invasive diagnostic technique for the early identification of TCS abuse on facial skin. In cases of TSDF, it serves multiple purposes, ranging from confirming the diagnosis to distinguishing it from other causes of facial redness, and estimating the approximate duration of TCS abuse. Additionally, dermoscopy may also aid in evaluating disease severity earlier than examination with the naked eye alone. Furthermore, it can assist in counseling patients with the aid of dermoscopic images for better insight.
